# Codon Usage Patterns in *Corynebacterium glutamicum*: Mutational Bias, Natural Selection and Amino Acid Conservation

**DOI:** 10.1155/2010/343569

**Published:** 2010-04-22

**Authors:** Guiming Liu, Jinyu Wu, Huanming Yang, Qiyu Bao

**Affiliations:** ^1^James D. Watson Institute of Genome Sciences, Zhejiang University, Hangzhou, Zhejiang 310008, China; ^2^Zhejiang Provincial Key Laboratory of Medical Genetics, Institute of Biomedical Informatics, Wenzhou Medical College, Wenzhou, Zhejiang 325000, China; ^3^Beijing Genomics Institute, Chinese Academy of Sciences, Beijing 101300, China

## Abstract

The alternative synonymous codons in *Corynebacterium glutamicum*, a well-known bacterium used in industry for the production of amino acid, have been investigated by multivariate analysis. As *C. glutamicum* is a GC-rich organism, G and C are expected to predominate at the third position of codons. Indeed, overall codon usage analyses have indicated that C and/or G ending codons are predominant in this organism. Through multivariate statistical analysis, apart from mutational selection, we identified three other trends of codon usage variation among the genes. Firstly, the majority of highly expressed genes are scattered towards the positive end of the first axis, whereas the majority of lowly expressed genes are clustered towards the other end of the first axis. Furthermore, the distinct difference in the two sets of genes was that the C ending codons are predominate in putatively highly expressed genes, suggesting that the C ending codons are translationally optimal in this organism. Secondly, the majority of the putatively highly expressed genes have a tendency to locate on the leading strand, which indicates that replicational and transciptional selection might be invoked. Thirdly, highly expressed genes are more conserved than lowly expressed genes by synonymous and nonsynonymous substitutions among orthologous genes fromthe genomes of *C. glutamicum* and *C. diphtheriae*. We also analyzed other factors such as the length of genes and hydrophobicity that might influence codon usage and found their contributions to be weak.

## 1. Introduction

It is well established that the codon usage patterns are generally not used with equal frequency. Grantham et al. firstly explained the phenomena of unequal usage and proposed the “genome hypothesis”, stating that the biases are species specific [1], and multivariate analysis methods were used to analyze codon usage and amino acid composition [2–4]. As more and more complete genome sequences of diverse species are investigated, researchers have found that biased usage of synonymous codons may result from various factors. Some unicellular species have extremely biased compositions, where compositional constraints are the main factors in determining the codon usage variation among genes [5–7]. In contrast, both translational selection and compositional constraint operate on the codon usage variation in other organisms [8–14]. Moreover, in several bacteria, the replication and translational selection is responsible for the codon usage variation among genes [15–18]. In organisms, such as *Escherichia coli *[36],* Drosophila melanogaster *[19], and *Caenorhabditis elegans* [20], the frequency of codon usage is directly proportional to the corresponding tRNA population and the preferred codons in highly expressed genes are recognized by the most abundant tRNAs. Meanwhile, it has been reported that amino acid conservation and hydrophobicity are the main factors shaping codon usage among the genes in *Mycobacteria* [21, 22]. Other factors may also influence the synonymous codon usage, such as protein secondary structure [23–26], mRNA folding stability [27, 28], gene function [29, 30], and gene length [31–33]. 

*Corynebacterium glutamicum* ATCC 13032, used industrially for the production of amino acids, is an aerobic, gram-positive rod-shaped bacteria capable of growing on a variety of sugars or organic acids [34]. In this study, we used the available complete genome sequence of this organism and analyzed its codon usage, aiming to understand the genetic organization of the *C. glutamicum *genome. Our results show that mutational bias, natural selection, and amino acid conservation are the main factors driving codon usage patterns in *C. glutamicum* gene*s.*

## 2. Materials and Methods

### 2.1. Genome Sequence Data

The complete genome sequences and coding sequences of* C. glutamicum* and *C. diphtheriae* were obtained from the NCBI ftp site (ftp://ftp.ncbi.nih.gov/genomes/Bacteria/). To minimize sampling errors, only genes of at least 100 codons in length with correct initiation and termination codons were used in further analysis.

### 2.2. Multivariate Analysis of Codon Usage

The COA (codon usage correspondence analysis, plots the codon usage data in a multidimensional space of 59 axes, excluding Met, Trp, and termination codons, identifies the axes which represent the most prominent factors contributing to the variation among genes), GC3s (the frequency of G + C at the third synonymously variable coding position, excluding Met, Trp, and termination codons), ENC (the “effective number of codons”; a measure of the bias in codon usage of genes, usually highly expressed genes display lower values compared with lowly expressed ones), RSCU (the “relative synonymous codon usage”; a value greater than 1.0 indicates that the corresponding codon is more frequently used than expected, whereas the reverse is true for RSCU values less than 1.0), CAI (the “codon adaptation index”; high values mean higher codon usage bias and higher expressed level), Fop (the “frequency of optional codons”), GRAVY index of hydrophobicity, and A3s, G3s, C3s, and T3s (the composition of each individual base A, G, C, and T at the third synonymous codon positions) were performed using the program CodonW1.42 (http://codonw.sourceforge.net/).The CAI was calculated taking the codon usage of the ribosomal proteins as a reference. Other statistical analyses were performed with the SPSS statistical software version 11.0.

### 2.3. Locating Genes Situated on the Leading and Lagging Strands of Replication

Asymmetrical mutational bias between the two complementary strands may contribute to variations in codon usage. To locate the genes on the leading or lagging strand of replication, the sites of origin and termination were determined by using the oriloc program (ftp://pbil.univlyon1.fr/pub/logiciel/oriloc/oriloc.c) and GC skew (G − C/G + C) was determined using the GC Skewing program (http://www.genomicsplace.com/gc_skew/gc_skew.html) by taking a 24 kb window size and a step size of 3 kb to locate the leading and lagging strands.

### 2.4. Orthologous Gene Pairs and Analysis

Orthologous genes were identified by the reciprocal best blast hit approach as those pairs displaying value of 60% identity, an E-value of 10^−5^, and overlapped by at least 60% of the length of the longest protein, with at least 100 amino acids in length using the local BLASTP program (http://www.ncbi.nlm.nih.gov/BLAST/download.shtml). The protein sequences of 1525 orthologous gene pairs were aligned using the MUSCLE program (http://www.drive5.com/muscle); then the aligned protein sequences were used to generate the corresponding codon alignment. The Ka (the number of synonymous substitutions per site) and Ks (the number of nonsynonymous substitutions per site) for each pair of aligned sequences were estimated using the PAML version 4.3 package (http://abacus.gene.ucl.ac.uk/software/paml.html) with runmode = −2 and CodonFreq = 2. Only those pairs of sequences having Ks values below 1.0 were considered in further analysis and the final dataset was comprised of 437 gene pairs.

## 3. Results

### 3.1. Overall Codon Usage

**Figure 1:**
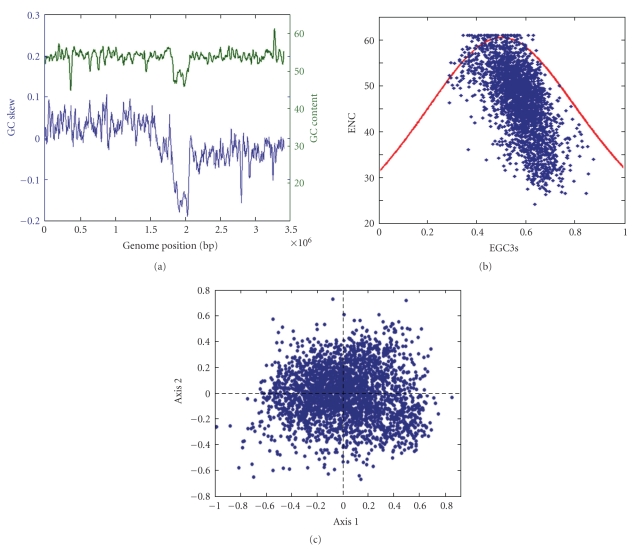
(a) The GC content and GC skew of the genome *C. glutamicum* with a 24 kb of window size and a 3 kb of step size. (b) The ENC plot of *C. glutamicum*. The continuous curve represents the relationship between GC3s and ENC values under random codon usage. (c) Distribution of *C. glutamicum *genes on the plane defined by the two main axes of the correspondence analysis.

As shown in Figure 1(a), the genome of *C. glutamicum* is biased towards high G + C contents ranging from 40% to 68% with an average of 54.7% and a standard deviation of 3.7%. With the exception of small regions, its genome shows little variation around the mean value. Due to composition constraints, G and C are expected to predominate at the third position of codons. Indeed, the codon usage indicated that C ending codons are predominant overall (data not shown). In order to understand the codon usage variation among different genes, ENC and GC3s values were calculated (Figure 1(b)). ENC values vary from 24.46 to 61.00 with a mean of 46.9 and standard deviation of 7.55%. The heterogeneity of codon usage was further confirmed from the GC3s values ranging from 28% to 87% with a mean of 57.18% and standard deviation of 8.3%. Wright suggested that plotting ENC against GC3s values could be used to effectively explore codon usage variation among genes [35]. If GC3s are the only determination of the codon usage variation among genes, then the values of ENC would fall on the continuous curve. The GC3s versus ENC plot reveals that only a small proportion of points lie on the expected curve (Figure 1(b)), which indicates that apart from the effect of compositional constraints, there might be some additional factors driving codon usage variation among the genes.

### 3.2. Gene Expression and Codon Usage Bias

In order to investigate the other possible trends in shaping codon usage variation among the genes in *C. glutamicum,* we subjected the data to multivariate statistical analysis. Figure 1(c) shows the position of genes along the first two axes. At the positive end of the first axis, it comprises of putatively highly expressed genes, such as ribosomal proteins, translation elongation factors, while the majority of putatively lowly expressed genes are scattered towards the other extreme. A more important result emerged when the genes were sorted according to their respective CAI values, and the highest positions were displayed not only by the genes encoding ribosomal protein but also by almost the same genes along the extreme of the first axis. Table 1 shows the first axis accounts for 20.33%, compared with 10.5% of the second axis and this value of the first axis is high and much larger than that of the second axis, indicating a primary trend in codon usage across genes. Furthermore, there are positive correlations between the first axis and CAI (*r* = 0.855, *P* < .001), with Fop (*r* = 0.892, *P* < .001), with GC3s (*r* = 0.594, *P* < .001), and especially with C3s (*r* = 0.881, *P* < .001). Those results suggest that gene expression may be the main factor shaping the codon usage in this organism, the first axis is associated with expression levels, and highly expressed genes have higher (G + C) content, especially C content at their synonymous third codon position than lowly expressed genes.

To investigate the differences between highly and lowly expressed genes, we compared the codon usage of genes that locate the two extremes of the first axis (Table 2). Chi square tests were performed taking *P* < .01 as the significant criterion. We found that there were 22 coding codons (corresponding to 18 amino acids) that are more highly used in putatively highly expressed genes than putatively lowly expressed genes. Among the 20 codons, there are 14 C ending codons and 3 G ending codons, which demonstrate that the presumed highly expressed genes tend to be C3-rich.

### 3.3. Replicational and Transcriptional Selection and Codon Usage

Recent reports of several bacterial strains show that codon usage bias is mainly governed by transcriptional and translational selection [19, 20, 36, 37]. After the origin versus termination and leading versus lagging strands were determined, we located the genes on the leading or lagging strands of replication and found that the proportion of genes located on the leading strands increases with CAI, from about 55% for low CAI genes (<0.35) to 67% for high CAI genes (>0.65) in the organism. For the putatively highly expressed ribosomal proteins, the proportion of genes on the leading strands reaches 84% (44/52) (Table 3). This observation is consistent with previous research results that essential genes are enriched to a greater extent than nonessential genes in the leading strand [38].

### 3.4. Gene Conservation and Codon Usage

**Figure 2:**
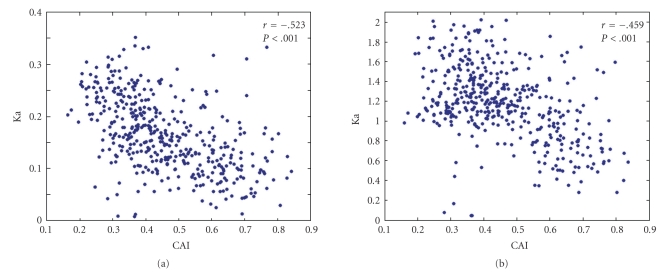
Plot of CAI values for *C. glutamicum *against Ka and Ks. (a) Plot of CAI values for *C. glutamicum *against Ka. (b) Plot of CAI values for *C. glutamicum *against Ks. The correlation coefficients (*r*) and level of significance (*P*) are shown.

The rate of synonymous substitutions has been reported to be nonuniform among different genes in the same species [39]. When we calculated the Ka and Ks between the orthologous genes from *C. glutamicum* and *C. diphtheriae*, several results were determined. Firstly, there is a negative correlation between the Ka and CAI value (*r* = −0.523, *P* < .001), comparative with Ks and CAI values with *r* = −0.459 and *P* < .001 (Figure 2). When the genes are sorted according to the respective Ks, the genes displaying the lowest values are those presumed highly expressed genes, such as ribosomal protein and translation elongation factors., Taken together, this indicates that highly expressed genes have diverged less at the synonymous position than lowly expressed genes. Secondly, the Ka and Ks are correlated with *r* = 0.473, *P* < .001. Thirdly, there is significant correlation between Ks and Fop (*r* = −0.431, *P* < .001), which indicated that the genes diverging less are the ones displaying highest frequencies of optional codon usage. 

Finally, we also investigated the relationship between codon usage and gene length (*r* = −0.137, *P* < .001), codon usage, and hydrophobicity (*r* = −0.094, *P* < .001), suggesting that their contributions to the codon usage variation are weak.

## 4. Discussion

Among prokaryotes, it is generally accepted that the preferences of synonymous codons can be explained as the result of mutational bias and natural selection acting at the level of translation. In *C. glutamicum*, the composition bias towards GC constraint indicates that these bases are predominant at the third codon positions across all genes. Indeed, the putatively highly expressed genes show an increment of several codons, most of which are C-ending triplets. Ikemura showed that there is a match between these codons and the most abundant tRNAs [36]. In *Escherichia coli* [36],* Drosophila melanogaster* [19], and *Caenorhabditis elegans* [20], highly expressed genes have a strong selective preference for codons with a high concentration for the corresponding acceptor tRNA molecule; the preferred codons are those best recognized by the most abundant tRNAs. This trend has been interpreted as the coadaptation between amino acid composition of protein and tRNA-pools to enhance the translational efficiency. Remarkably, in this study, there is a strong positive correlation (*r* = 0.94, *P* < .001) between the Fop in each gene and respective CAI value. This strongly suggests that translational selection influenced the codon usage of *C. glutamicum* and the “optional codons” were more frequent in highly expressed genes. 

As more prokaryotic genomes are analyzed, it becomes evident that codon usage is rather dependent on mutational bias and natural selection. For example, the complex pattern of codon usage in *Chlamydia trachomatis* is inferred to be the result of strand-specific mutation, natural selection, the hydropathy level of each protein and amino acid conservation [17]. In this study, we present evidence suggesting that, apart from mutational bias and natural selection, strand-specific and amino acid conservation also contribute to the codon usage of *C. glutamicum*. Strand bias also dominates codon usage in other symbiotic or parasitic bacteria, such as *Rickettsia prowazekii*,* Borrelia burgdorferi, *and *Lawsonia intracellularis* [15, 40, 41]. We found a distribution bias of genes (particularly for those with a high CAI) on the leading strands in *C. glutamicum*. This is usually interpreted as the result of “replicational selection”, by which presence on the leading strand would permit the avoidance of collision between polymerases when replication and transcription occur at the same time [16]. 

It was reported that the codon usage is more biased for amino acid that are more conserved between species [42, 43]; natural selection has a larger contribution than mutation to the observed correlation between evolutionary rates and gene expression level in *Chlamydomonas* [44]. A correlation between Ks and Fop was also identified. This correlation with Ka might be explained in many ways. Akashi argued that the selection for translation accuracy maintains a high frequency of preferred codons for highly conserved amino acids [43]. Two additional hypotheses for this pattern are a possible mechanistic bias in mutation and the fact that synonymous sites are also subject to some degree of selection [45]. The latter scenario could mean either selection on codon usage, or that synonymous substitutions might not always be silent or evolutionary responses to adaptations [46]. A similar interaction between the level of expression, the level of codon bias, and gene conservation was demonstrated in *Mycobacterium* [21].

In summary, this study has shown that the codon usage variation among the genes of *C. glutamicum* is influenced by mutational bias, translational selection, and amino acid conservation. As more complete prokaryotic genomes are being studied, different factors shaping the pattern of codon usage might be found.

## Figures and Tables

**Table 1:** Result of factorial correspondence analyses on codon usage in *C. glutamicum. *

	Interia	CAI	GC3s	G3s	C3s	A3s	T3s
Axis1	20.33	0.855**	0.594**	0.557**	0.881**	−0.112**	−0.593**
Axis2	10.49	0.367**	0.006	0.524**	0.336**	0.657**	−0.542**

**Correlation is significant at the 0.01 level.

**Table 2:** Codon usage in putative highly expressed and lowly expressed genes of C. *glutamicum. *

AA	Codon	High		Low		AA	Codon	High		Low	
		*N*	RSCU	*N*	RSCU			*N*	RSCU	*N*	RSCU
Phe	UUU	149	0.17	1058	1.37	Ser	UCU	371	0.77	539	1.22
	UUC*	1608	1.83	481	0.63		UCC*	1972	4.11	284	0.64
Leu	UUA	19	0.03	486	0.78		UCA	175	0.36	360	0.82
	UUG	239	0.33	1284	2.06		UCG	59	0.12	676	1.53
	CUU	656	0.91	707	1.13	Pro	CCU	527	0.92	492	1.11
	CUC*	1583	2.21	341	0.55		CCC	185	0.32	205	0.46
	CUA	140	0.2	274	0.44		CCA*	1454	2.55	371	0.84
	CUG*	1666	2.32	651	1.04		CCG	119	0.21	705	1.59
Ile	AUU	412	0.43	1207	1.71	Thr	ACU	304	0.38	605	1.2
	AUC*	2477	2.57	654	0.93		ACC*	2758	3.45	427	0.85
	AUA	7	0.01	257	0.36		ACA	74	0.09	378	0.75
Met	AUG	1143	1	863	1		ACG	61	0.08	601	1.2
Val	GUU*	1495	1.45	883	1.11	Ala	GCU*	1707	1.18	957	1.08
	GUC*	1631	1.59	452	0.57		GCC*	1138	0.79	512	0.58
	GUA	336	0.33	396	0.5		GCA*	2529	1.75	789	0.89
	GUG	650	0.63	1443	1.82		GCG	411	0.28	1300	1.46
Tyr	UAU	47	0.07	632	1.41	Cys	UGU	64	0.44	213	1.3
	UAC*	1311	1.93	262	0.59		UGC*	230	1.56	114	0.7
TER	UAA	100	2.19	39	0.85	TER	UGA	4	0.09	47	1.03
	UAG	33	0.72	51	1.12	Trp	UGG	577	1	661	1
His	CAU	47	0.09	560	1.35	Arg	CGU	744	1.62	557	1.45
	CAC*	981	1.91	271	0.65		CGC*	1887	4.11	362	0.94
Gln	CAA	284	0.34	539	0.79		CGA	89	0.19	317	0.83
	CAG*	1385	1.66	830	1.21		CGG	16	0.03	489	1.27
Asn	AAU	141	0.13	795	1.37	Ser	AGU	19	0.04	483	1.09
	AAC*	1955	1.87	369	0.63		AGC	284	0.59	305	0.69
Lys	AAA	298	0.26	664	0.91	Arg	AGA	5	0.01	236	0.62
	AAG*	2014	1.74	800	1.09		AGG	13	0.03	341	0.89
Asp	GAU	959	0.55	1537	1.53	Gly	GGU	1026	0.93	1069	1.46
	GAC*	2559	1.45	468	0.47		GGC*	2830	2.55	562	0.77
Glu	GAA*	2117	1.04	1095	0.91		GGA	549	0.5	568	0.78
	GAG	1941	0.96	1313	1.09		GGG	26	0.02	724	0.99

*N*: the number of codons; AA: amino acid.

*Codon with significantly (*P* < .01) higher frequencies in highly expressed genes.

High: codons in highly expressed genes; Low: codons in lowly expressed genes.

**Table 3:** Percentages of genes in *C. glutamicum* on the leading (versus lagging) strand.

Range	Total number	Leading		Lagging	
	*n*	*n*	Percent	*n*	Percent
CAI < 0.35	1958	1087	55.52	871	44.48
0.35 < CAI < 0.65	721	434	60.19	287	39.81
CAI > 0.65	61	41	67.21	20	32.79
Ribosomal	52	44	84.62	8	15.38
